# Assessing and recovering Alzheimer’s disease: a comparative analysis of standard neuropsychological approaches and virtual reality interventions with the use of digital storytelling

**DOI:** 10.3389/fpsyg.2024.1406167

**Published:** 2024-07-24

**Authors:** Fabrizio Stasolla, Mariacarla Di Gioia, Irene Messina, Francesco Treglia, Anna Passaro, Antonio Zullo, Mirella Dragone

**Affiliations:** ^1^Faculty of Law, Giustino Fortunato University, Benevento, Italy; ^2^Universitas Mercatorum, Rome, Italy; ^3^Academy of Mind Ecology-School of Specialization in Systemic Relational Psychotherapy, Rome, Italy

**Keywords:** Alzheimer’s disease, assessment, cognitive rehabilitation, virtual reality, digital storytelling

## Abstract

**Background:**

Alzheimer’s disease (AD), the most common form of dementia, is a progressive neurodegenerative disorder that predominantly affects the elderly population. Traditional assessment methods, including neuropsychological tests like the MMSE, have been the cornerstone of AD diagnosis for decades. These methods are grounded in a wealth of research and clinical experience, providing a robust framework for understanding the cognitive deficits of AD. The evolution of AD assessment and rehabilitation has recently been tackled with the introduction of Virtual Reality (VR) technologies.

**Objectives:**

To evaluate the use of storytelling and reminiscence therapy in virtual reality programs as a complementary and enhancing modality alongside standard assessment and rehabilitation for Alzheimer’s patients. To explore how regular interaction with VR narratives can slow cognitive decline or improve relevant features of cognitive functioning over the time. To propose a new assessment and rehabilitative tool based on the use of VR and digital storytelling.

**Method:**

A comparative analysis of Standard Neuropsychological Approaches and Virtual Reality Interventions in patients with Alzheimer disorder was carried out. A literature overview on the empirical studies between 2019 and 2024 was conducted.

**Results:**

We propose a new VR-based setup mediated by the use of storytelling for the assessment and recovery of AD.

**Conclusion:**

The employment of storytelling within VR programs for the assessment and rehabilitation of Alzheimer’s disease can positively impact both the cognitive and emotional realms of patients, with beneficial outcomes on caregivers’ and families’ burden. The successful implementation of this approach requires careful consideration of accessibility, data interpretation, and standard validation protocols.

## 1 Introduction

Alzheimer’s disease (AD) is characterized by a gradual impairment in cognitive functions. It leads to significant memory loss, language deterioration, impaired visuospatial skills, and executive dysfunction. The pathological hallmarks of Alzheimer’s include the deposition of amyloid-beta plaques and tau protein neurofibrillary tangles in the brain, contributing to neuronal loss and brain atrophy (American Psychiatric Association, 2013). Exacta comprehensive etiology of AD remains an area of intensive research, with a general consensus pointing toward a multifactorial origin encompassing genetic, environmental, and lifestyle factors. Specifically, Jingjing et al. (2022) identified 69 proteins with genetically predicted concentrations showing associations with AD risk.

The neuropsychological assessment of patients with Alzheimer’s disease is a complex process that aims to identify and quantify the cognitive and behavioral deficits associated with the pathology. A crucial feature of neuropsychological assessment is the ability to differentiate between normal aging processes and pathological cognitive declines. The neuropsychological dimension plays a crucial role in the management of AD, from initial assessment to the planning and implementation of rehabilitative interventions (Weintraub et al., 2012).

In the last decades, virtual reality (VR) technology is emerging as a novel tool in the assessment and rehabilitation of Alzheimer’s disease. Virtual technology, combined with traditional methods such as exercise therapy, provides new insights for innovative cognitive evaluation and intervention. For example, VR-based setups have evidenced considerable detection performance in detecting mild cognitive impairment (MCI). Accordingly, VR-based setups can serve as recommended screening methods (Lu et al., 2023; Yu et al., 2024). Recent studies (Varela-Aldás et al., 2022; Tortora et al., 2024) explored the use of advanced technologies, such as virtual reality and augmented reality systems, in the cognitive rehabilitation of Alzheimer’s patients. Tortora et al. (2024) reviewed VR-based cognitive rehabilitation therapy (VR-CRT) as a paramount in treating MCI for its additional ecological and adaptive advantages. Their review highlighted that VR-based setups were effective as the cognitive rehabilitation therapy (CRT) for all the outcome measures recorded.

Varela-Aldás et al. (2022) investigated the potential of an immersive virtual reality (IVR) application (i.e., the Cupboard task) for the evaluation of memory in a more ecological way using a daily living activity. Results endorsed the use of VR in clinical settings for cognitive training and supported cognitive health of MCI or AD.

VR setups provide immersive and interactive environments useful to assess cognitive functions in a more dynamic and ecologically valid way. Furthermore, VR enhances rehabilitative purposes, providing tailored cognitive exercises aimed at fostering constructive engagement enhanced by cognitive reserve (Caffò et al., 2016). Consequently, the progression of cognitive decline should be reduced. Specifically, VR–based setups can provide remote monitoring and intervention, making it a valuable tool in the context of increasing prevalence and the need for scalable solutions in Alzheimer’s care (Bayahya et al., 2019; Bernini et al., 2021; Stasolla et al., 2021). VR also offers a multisensory experience that can be effective for Alzheimer’s patients, as it simultaneously stimulates multiple sensory channels, thus improving information processing and memorization. Moreno et al. (2019) demonstrated that VR-based interventions used in cognitive rehabilitation were helpful in improving cognitive functioning (i.e., memory, dual tasking, and visual attention) and psychological support (i.e., reduction of anxiety, higher levels of well-being, and increased use of coping strategies). In an umbrella review comprising meta-analyses of randomized controlled trials, Yu et al. (2024) concluded that the VR technology in older people with cognitive disorders improved only specific cognitive abilities.

Baldimtsi et al. (2023) investigated the effects of a VR Training System, named VRADA (VR Exercise App for Dementia and Alzheimer’s Patients), on the cognitive functioning of older people with MCI. Specifically, the VRADA system was developed to motivate and encourage MCI patients to exercise physically and cognitively via a user-friendly, effective, and safe system. VRADA system improved the cognitive function of elders with MCI. Therefore, VR-based setups offer new opportunities for creating stimulating and controlled environments in which patients can safely practice daily activities, thus enhancing their cognitive and functional abilities. Virtual technology, combined with traditional methods such as exercise therapy, provides new insights for innovative cognitive evaluation and cognitive intervention (Zhu et al., 2024). Matsangidou et al. (2023) compared the conventional physical training that people living with Dementia (PwD) received in a nursing home against Semi Immersive VR (SIVR) and Fully Immersive VR (FIVR) training paradigms. Data indicated that FIVR can improve the training of PwD, leading to more accurate execution of the exercises and preventing external distractions.

The incorporation of digital storytelling into VR programs for the assessment and rehabilitation of Alzheimer’s patients can represent an innovative approach that leverages narrative power combined with immersive technologies to create meaningful and engaging therapeutic experiences (Rios Rincon et al., 2022).

Fels and Astell (2011) argued on storytelling as a conversation model for people with dementia. This methodology emerged as a new strategy, offering unique benefits in the context of neuropsychology and making a significant contribution in AD management and clinical practice. Hollinda et al. (2023) claimed for communication, relational, and technological elements of digital storytelling as crucial means of facilitators to create digital stories for persons living with dementia. Digital storytelling was a meaningful activity for persons living with dementia to reinforce one’s identity (Park et al., 2017).

Rios Rincon et al. (2022) carried out a systematic review to examine the range and extent of the use of digital technologies for facilitating storytelling in older adults and their care partners. Data indicated that, although the level of evidence of its effectiveness was low, the most common use of digital storytelling supported older adults’ memory, reminiscence, identity, and self-confidence. Stargatt et al. (2022) showed that digital storytelling was promising as an effective approach for supporting well-being in older adults.

Sljivic et al. (2022) carried out research into the use of digital stories containing narratives from older adults and its ability to evoke empathy. It showed a positive change in participants’ attitudes post-viewing compared to pre-viewing the digital stories.

In light of the above, the objectives of the current perspective paper were (a) to propose the use of digital storytelling and reminiscence therapy in VR programs as a complementary and enhancing modality alongside standard assessment and rehabilitation, (b) to evaluate its effects on emotional and cognitive empowerment of AD, and (c) to assess the secondary outcome on caregivers’ and families’ burden reduction.

The following sections detail the assessment and rehabilitative comparison between standard neuropsychological tests and VR-based setups and digital storytelling, an overview on the use of digital storytelling in older adults, and a perspective proposal as a complementary strategy compared to neuropsychological standard assessment. Finally, we critically discussed practical implications for daily settings. Limitations and future research perspectives were additionally highlighted.

### 1.1 Assessment and cognitive rehabilitation of Alzheimer’s disease: standard vs. VR approaches

The comprehensive assessment of AD through traditional methods is a multi-layered process, pivotal for accurate diagnosis and treatment planning. This process goes beyond mere symptom identification, delving into a thorough exploration of the patient’s cognitive, emotional, and functional capabilities (Deary et al., 2009; McKhann et al., 2011; Arevalo-Rodriguez et al., 2021; Creavin et al., 2022; Bradfield, 2023; Harris-Gersten et al., 2023). The assessment commonly has an onset with structured interviews, including detailed clinical histories that involve not only the patient but also family members or caregivers. These interviews are purposeful uncover the initial decline, progression, and impact of symptoms in daily living. They also explore familial history, as genetics play a significant role in AD. Clinicians may use standardized tools scale to systematically capture this information, also to implement prevention strategies (Tsoi et al., 2015; Lisko et al., 2021; Lissek and Suchan, 2021; Festari et al., 2023).

The integration of VR in the assessment of AD represents a significant technological advancement in neuropsychology (Coelho et al., 2020; Jonson et al., 2021; Kim et al., 2022; Gómez-Soria et al., 2023). These systems provide a fully immersive experience, allowing patients to interact within a three-dimensional environment. They have been used to simulate real-life scenarios and tasks to assess cognitive functions such as memory, spatial orientation, and executive functions in a more ecologically valid setting compared to traditional neuropsychological tests. For example, Cabinio et al. (2020) showed similar discriminating abilities for smart aging serious game (SASG) and gold standard tests, and a greater discrimination ability compared to non-specific neuropsychological tests. They revealed that the SASG outperformed the Montreal Cognitive Assessment test (MoCA) in the ability to detect neuronal degeneration in the hippocampus on the right side. SASG allowed the early assessment of cognitive impairment through ecological tasks and potentially in a self-administered way (Isernia et al., 2021). Relevant studies compared the diagnostic accuracy of VR-based assessments with traditional neuropsychological tests (Arevalo-Rodriguez et al., 2015; Tariot et al., 2024). Those contributions commonly found that VR accurate includes the daily living cognitive abilities of AD patients (Tarnanas et al., 2013). Specifically, Chua et al. (2019) demonstrated the feasibility of using a VR-based screening tool for cognitive function in older persons in primary care. According to Cogné et al. (2017), unlike pencil-and-paper tests, VR is useful to assess large-scale navigation strategies in patients with brain injury or schizophrenia, or in the context of dementia.

Cognitive rehabilitation for AD has traditionally focused on a range of therapies designed to slow cognitive decline, manage symptoms, and improve quality of life. These conventional methods usually include cognitive training, cognitive stimulation, and various forms of psychotherapy (Vega and Newhouse, 2014). Such methods involve structured activities aimed at improving specific cognitive functions such as memory, attention, and problem-solving skills. Techniques like repetition, mnemonic devices, and problem-solving exercises are commonly targeted (Cicerone et al., 2019; Germain et al., 2019; Fadhi et al., 2023; Krellman and Mercuri, 2023). The aforementioned techniques include approaches such as reminiscence therapy (Bayram, 2024), in which patients are encouraged to recall past experiences, and occupational therapy (Smallfield et al., 2024), which involves repeated exposure to information about time, place, or person to improve understanding of their environment. Group activities, social interaction, and behavioral management techniques are also important features of traditional rehabilitation (Fetherstonhaugh et al., 2019).

VR-based interventions have recently emerged as a novel approach. These involve the use of immersive VR technology to create simulated environments in which patients can interact. These environments can be tailored to replicate real-life scenarios or to create therapeutic settings that engage specific cognitive functions (Muurling et al., 2023; Catania et al., 2024). VR-based interventions showed encouraging results, specifically in engaging multiple cognitive domains simultaneously. Moulaei et al. (2024) conducted a study in which VR-based interventions have demonstrated positive outcomes in enhancing cognitive functioning and addressing cognitive impairment. In a systematic review, Kim et al. (2022) concluded that feedback stimulation through VR had a potential value in improving cognitive functioning of individuals with MCI.

Ren et al. (2024) detected that VR-based rehabilitation training was a beneficial nonpharmacologic approach for managing MCI or dementia. They concluded that immersive VR-based training had greater effects on cognition and motor function than non-immersive VR-based training, but non-immersive VR-based training was more convenient for patients with limitations imposed by their disease.

Zhong et al. (2021) showed that VR cognitive training might be beneficial for improving global cognitive functions and executive functions in individuals with MCI, although the effects were in the short term. Gómez-Soria et al. (2023) also suggested that VR-based interventions were beneficial for improving cognitive functioning in patients with MCI.

In a mini-review, D’Cunha et al. (2019) detected that the use of virtual and augmented reality technology for people living with dementia (PLWD) and MCI was a novel and emerging method which could provide cognitive stimulation and improve well-being.

With regard to the outcomes, the aforementioned studies evidenced that VR-based cognitive rehabilitation can lead to improvements in cognitive functions similar to those achieved through traditional methods.

### 1.2 Digital storytelling in VR for cognitive rehabilitation

Storytelling has longly been recognized as a powerful medium for communication and learning. In the context of AD, storytelling can particularly be effective in cognitive rehabilitation (Howarth, 2020; Kim, 2023; Biskupiak et al., 2024). The use of storytelling in cognitive rehabilitation is grounded in several psychological theories. For instance, narrative psychology suggests that storytelling is a critical human strategy for making sense of the world (Merrilees et al., 2023). It implies that narratives help in organizing and retaining information, which is crucial for individuals with cognitive impairments (Moss and Björn, 2006). Furthermore, the Social Cognitive Theory (Kindell et al., 2017) posits that observing others in stories can lead to learning and behavior changes, which can be beneficial in cognitive rehabilitation.

Ma et al. (2023) conducted a meta-analysis on the effectiveness of creative story therapy versus routine nursing alone for the treatment of dementia. They found that creative story therapy combined with routine nursing had significant effectiveness in improving cognitive functioning and depression in people with dementia. Vigliotti et al. (2019) evaluated the benefits of TimeSlips, a group creative storytelling intervention used in residential care settings, on quality of life (QOL), interactions with caregivers, and Mini-Mental State Examination (MMSE) scores for persons with varying degrees of dementia severity. They evidenced that participants initially classified with mild–moderate dementia were significantly more likely to experience positive benefits compared to those initially classified with severe dementia.

The integration of storytelling into VR environments represents a novel approach, blending the immersive nature of VR with the narrative power of storytelling. Storytelling combined with VR can stimulate both memory and emotional engagement. VR storytelling can incorporate spatial and temporal cues that aid in orientation, commonly challenged in AD. By situating the narrative in recognizable and relatable environments, patients can practice navigational skills and temporal orientation. Personalized stories, or those that are culturally and historically relevant to the patient, can evoke autobiographical memory, which is the key in maintaining a sense of identity in AD patients (Stargatt et al., 2022).

Huang and Yang (2022) emphasized that immersive VR reminiscence can improve mood and preserve cognitive functions in older patients with dementia during the intervention. Emotional engagement in a story can also enhance cognitive processing and retention of information.

Khirallah Abd El Fatah et al. (2024) investigated the effect of VR reminiscence versus traditional reminiscence therapy (RT) on cognitive function and psychological well-being among older adults in assisted living facilities. They concluded that application of VR reminiscence or traditional RT was efficacious in improving cognitive functioning and psychological well-being among institutionalized older adults. Therefore, the employment of digital storytelling within VR programs for the assessment and rehabilitation in AD significantly impacted both the cognitive and emotional realms of patients, while also positively affected caregivers’ burden.

## 2 A perspective proposal

We conducted a search in Scopus, merging the keywords “Digital Storytelling” and “Alzheimer disease,” which resulted in a pool of 3 documents with specific studies conducted from 2019 to 2024 (da Silva et al., 2022; Hollinda et al., 2023; Xu et al., 2023), excluding two reviews (Rios Rincon et al., 2022; Stargatt et al., 2022; see Table 1).

**Table 1:** Reviewed contributions between 2019 and 2024 on the use of digital storytelling in AD.

Authors	Participants	Objectives	Methodology	Main Outcomes
da Silva et al. (2022)	Ten community caregivers	Assessment of Caregivers perspectives on Communication	Semi-structured interviews	Caregivers appreciated the use of story telling as a crucial means for enhancing communication
Hollinda et al. (2023)	Nineteen caregivers and five facilitators	Exploring and Describing facilitation using storytelling	Qualitative Analysis	Communication, Relation, and Technology facilitated social interactions
Xu et al. (2023)	Ninety-Two diads composed by young and older adults	Investigating how reminiscence could improve social and emotional well-being	Randomized controlled trial	Intergenerational reminiscence was helpful in supporting communication
Rios Rincon et al. (2022)	Five hundred and ten participants	Examining the range and the extent of technology	Systematic Review	Low effectiveness in memory and reminiscence
Stargatt et al. (2022)	Sixty-two participants	Evaluating the methods for creating digital stories	Systematic Review	Promising approach in supporting well-being

Xu et al. (2023) suggested that reminiscence strategies combined with an intergenerational approach may yield significant social and mental health benefits for participants who have cognitive impairments. Hollinda et al. (2023) identified during digital storytelling facilitation with persons living with dementia, namely in: (a) communicating, (b) building collaborative relationships, and (c) using technology. Digital storytelling facilitators employed the three elements to weave together a person’s narrative with meaning.

Lastly, da Silva et al. (2022) detected that the use of Information and Communication Technologies (ICT) can provide support for communication between people living with dementia and their formal and informal caregivers, both locally and remotely. The aforementioned detailed literature, concisely summarized in Table 1, suggests that the use of digital storytelling can be helpful, effective, and suitable in reducing cognitive decline and promoting emotional interactions among AD population. Furthermore, empirical data support positive outcomes on families’ and caregivers’ burden reduction.

In line with the above, we proposed a new VR technological setup mediated by digital storytelling as a complementary strategy compared to standard neuropsychological assessment. By creating customized and tailored VR-based solutions, one can argue on the opportunity of differentiating between a normal cognitive decline due to aging and an early stage of dementia. Secondly, the later patients might be involved in further VR-based individualized environments aimed at supporting either cognitive or emotional dimensions. For example, Stasolla and Di Gioia (2023) proposed a technological setup based on reinforcement learning’s principles (RL). One can envisage the use of a combined option between RL and VR in which AD patients can be exposed to highly immersive and personalized environments constantly and continuously adapted to the participants’ performances (Khalid et al., 2024). The affordability and suitability in daily settings should be carefully assessed (Raj and Mirzaei, 2023). Based on different levels of AD, different technological solutions, environments, and setups may be implemented (Karana and Paun, 2023; Yi et al., 2023). For instance, for mild AD, one may rely on occupational or functional activities. For a moderate level of AD, one may support communication and/or leisure skills. For a severe to profound level of AD, one may promote reminiscence process or positive participation through music-based programs (Ardelean and Redolat, 2024). Finally, the integration of different technologies for supporting cognitive and emotional dimensions with a dual objective (i.e., assessment and rehabilitation) has recently been argued (Stasolla et al., 2023, 2024).

## 3 Discussion

We proposed a new VR setup mediated by digital storytelling as crucial means for assessing emotional and cognitive decline in aging. We hoped to differentiate between a normal decline and an early stage of dementia. Moreover, we planned a new customized rehabilitative approach for recovering emotional and cognitive decline. Tailored solutions based on RL principles should be outlined for providing highly individualized and updated options. The expected outcomes should include but are not limited to a constructive engagement, positive participation, emotional, and cognitive recovering (Bacanoiu and Danoiu, 2022; Jopowicz et al., 2022). The outcomes on caregivers’ and families’ burden can be addressed (Bernini et al., 2023; Petersen et al., 2023). Digital storytelling may be systematically be included. A combined approach between neuropsychological standard approach and VR-based setups mediated by storytelling and RL principles may be envisaged.

For example, Hollinda et al. (2023) differed from previous research as they discussed key elements of facilitating digital storytelling, rather than its benefits, limitations, or experiences, providing a new contribution to the literature. Accordingly, “facilitators” used communication strategies and relational skills to elicit information and build partnerships with participants: they used technology as a bridge to weave together participants’ narratives and meaning into a digital story as co-creators.

Xu et al. (2023) developed and tested reminiscence offered by trained young adult volunteers using a digital storytelling platform as a helpful tool for older adults affected by AD and related dementia to improve their social and emotional well-being. The authors showed that intergenerational reminiscence provided by young adult college student offered promising benefits for both the younger and older generations.

da Silva et al. (2022) suggested that either formal or informal caregivers were both concerned about how to understand and to be understood by the people they were caring for, and saw value in digital storytelling as a new kind of assistive communication tool for more visual communication. Specifically, the authors noted that new assistive communication technology could help with the expression of ideas through predictive speech or text options and visual stimuli. Furthermore, new assistive media technology such as digital storytelling tools could help caregivers facilitate storytelling and remembering in people with dementia, or capture joint activities in richer forms.

Therefore, the analyzed studies supported the new approach employing storytelling within VR programs for the assessment and rehabilitation of Alzheimer’s patients. Its use may significantly impact emotional aspects of patients, as well as their caregivers. Further research needs to be carried out to evaluate the impact of using digital storytelling on specific cognitive functions or domains in older adults with dementia.

## 4 Conclusion

The evolution of AD assessment and cognitive rehabilitation evidenced a significant shift with the introduction of VR technologies (Strong, 2020). The literature indicates that VR methods may show greater improvements in specific areas such as spatial awareness, attention, and executive functioning. Some studies also highlight the positive impact of VR-based rehabilitation on emotional well-being and quality of life (Appel et al., 2020; Chen et al., 2021).

The integration of storytelling into virtual reality programs for the assessment and rehabilitation of Alzheimer’s patients represents a cutting-edge in the field of neuropsychology and cognitive therapy. This approach exploits the ability of narratives to evoke memories, emotions, and social connections, amplifying the effectiveness of immersive technologies in the management of the disease, offering potential benefits in memory retention and quality of life. The analysis of digital storytelling showed how evoked long-term memories stimulated reminiscing brought mostly joy but occasionally moments of sadness to the persons with cognitive impairments, aided family members in remembering and better understanding their loved ones, or stimulated social interactions (Damianakis et al., 2010).

Zhu et al. (2024) conducted brainstorming sessions with people with MCI and their caregivers to identify the potential features of a digital storytelling application named Huiyou (“meeting new friends” in Chinese). They highlighted the potential of Huiyou to enhance well-being and facilitate meaningful social interactions while maintaining crucial existing relationships. Definitely, the employment of storytelling within VR programs for the assessment and rehabilitation of Alzheimer’s disease can significantly enhance both the cognitive and emotional realms of patients, while also positively affecting caregivers’ burden.

## 5 Limitations and future perspectives

The application of VR in distinguishing between normal aging and the potential development of dementia presents a novel and promising approach within the field of neuropsychological assessment and early intervention (Tian et al., 2023). VR technology, once integrated with storytelling and cognitive tasks, offers a unique and immersive platform for evaluating cognitive functions in a way that traditional assessments might not.

The proposed prospective toward using VR programs with storytelling in the assessment and rehabilitation of cognitive disorders represents a significant paradigm shift. This approach promises not only to enhance patient engagement and therapy personalization but also to offer a more comprehensive and effective means of cognitive stimulation. By leveraging the immersive and interactive capabilities of VR, coupled with the deep resonance of personal narratives, this innovative approach could redefine the standards of care in cognitive rehabilitation, offering patients a more engaging, effective, and meaningful path to recovery.

While the use of VR holds promise for enhancing the differentiation between normal aging and dementia, several challenges and considerations should be addressed:

- The effectiveness of VR as a tool may be influenced by individuals’ familiarity and comfort with technology. Older adults may require additional support and orientation to engage effectively with VR.
- The data obtained from VR assessments need to be interpreted within the broader context of an individual’s cognitive health, considering other medical, psychological, and social factors.
- There is a need for standardized protocols and validation studies to ensure that VR assessments accurately differentiate between normal aging and the early stages of dementia.
- We acknowledge that our integrated-based technology proposal currently lacks of empirical data supporting our claims. We are confident that future research will systematically incorporate that proposal.

The successful implementation of this approach with digital storytelling will require careful consideration of the challenges related to accessibility, technology availability, financial and human resources, data interpretation, and the need for standardized and validated assessment protocols (Tan et al., 2023).

## Author contributions

FS: Conceptualization, Writing – original draft. MDG: Conceptualization, Writing – original draft. IM: Writing – review & editing. FT: Writing – review & editing. AP: Supervision, Validation, Writing – review & editing. AZ: Writing – review & editing. MD: Writing – review & editing.
